# Nanomaterial-Based Optical Detection of Food Contaminants

**DOI:** 10.3390/foods13040557

**Published:** 2024-02-12

**Authors:** Mingfei Pan

**Affiliations:** State Key Laboratory of Food Nutrition and Safety, Tianjin University of Science and Technology, Tianjin 300457, China; panmf2012@tust.edu.cn

## 1. Introduction

The presence of food contaminants remains a significant aspect contributing to global food safety issues, drawing widespread attention from ordinary consumers, governments, and researchers [1,2,3]. These contaminants encompass various harmful factors, including residues of agricultural and veterinary drugs, biotoxins, heavy metals, allergenic proteins, and particularly endogenous hazardous substances generated during processing, representing a new focus in detection technology research [4,5].

Various nanomaterials with different structures and properties, such as metallic nanomaterials, up-conversion fluorescence nanomaterials, metal–organic framework porous materials, quantum dot fluorescence materials, etc., not only serve as solid carriers for bio-recognition elements (such as antibodies, aptamers, etc.) [6,7] and biomimetic recognition elements (such as molecularly imprinted polymers) [8,9], but also provide signal sources for visual, rapid, and convenient analysis [10,11,12]. Visible light, fluorescence, or electrochemiluminescence are quantitative signals commonly used in convenient food safety analysis strategies. This significantly promotes the development of precise and rapid analysis techniques for food contaminants, incorporating advanced methods such as nanomaterials, biomimetics and biorecognition, and chemometrics. Therefore, strategies based on multifunctional nanomaterials and utilizing antibodies, aptamers, and biomimetic polymers as recognition elements for the fluorescent or visual detection of contaminants in complex food matrices are gradually assuming crucial roles in food safety testing strategies [13,14,15].

## 2. An Overview of Published Articles

In the study by Tianyu Ma et al. (Contribution 1), the authors designed and prepared a zearalenone (ZEN) hapten against the mycotoxin ZEN, and the original coating ZEN-ovalbumin (ZEN-OVA) by conjugation with OVA. Based on gold nanorods (AuNRs) of uniform size and stable properties synthesized by the seed-mediated method, the indirect competitive enzyme-linked immunosorbent assay (ic-ELISA) and the AuNR growth-based multicolor ELISA for detecting ZEN toxin were further established. Under optimal experimental conditions, the coating amounts of ZEN-OVA were 0.025 µg/well, the antibody (Ab) dilution factor was 32,000 times, blocking solution was 0.5% skimmed milk powder, enzyme-labeled secondary Ab diluted 10,000 times, and at pH 7.4 of the PBS buffer, the sensitivity (*IC*_50_) of the established ic-ELISA for ZEN detection reached 0.85 ± 0.04 µg/L and the limit of detection (LOD, *IC*_15_) reached 0.22 ± 0.08 µg/L. In the multicolor ELISA based on the growth of AuNRs, as the content of ZEN increased, the mixed solution exhibited a significant color change from brownish red to colorless. ZEN concentrations as low as 0.1 µg/L could be detected with the naked eye (brown-red to dark gray). This study presents an effective analysis strategy for the rapid screening and accurate monitoring of ZEN contaminants in foods.

In the study by Lingyan Zhao et al. (Contribution 2), a novel rare earth upconversion nanomaterial with a three-layer sandwich core–shell structure was synthesized by an improved thermal decomposition method, and the morphology, fluorescence intensity, and diffraction peak position of the new material were characterized by TEM (transmission electron microscopy), XRD (powder X-ray diffraction), and fluorescence spectrophotometry. The inert core/active shell/inert shell design improved the upconversion luminous efficiency of the new material several-fold. FT-IR (Fourier transform infrared spectroscopy) characterization showed that the surface of activated upconversion nanoparticles was modified with the silicon shell and amino group. Combined with the characteristics that aminoated polystyrene magnetic microspheres could be separated by the magnetic field, an upconversion magnetic separation immunoassay method for the detection of pyrethroid pesticide residues was established. The capture probe competed with the pyrethroid standard, combined the signal probe, and measured the fluorescence signal value formed by the capture probe signal probe complex at 542 nm under 980 nm excitation light. The LOD of fenpropathrin was 0.01 µg/L, cypermethrin was 0.015 µg/L, and fenvalerate was 0.011 µg/L. Through the actual detection of apple, cabbage, and other samples, the recovery rate of pyrethroids was between approximately 83.4% and 97.8%. Comparison with the HPLC (high-performance liquid chromatography) detection results showed that the established method had good accuracy and could realize the quantitative analysis of pyrethroids in food.

In their research, Wenbo Zhu et al. (Contribution 3) coupled two upconversion materials with anti-clothianidin and anti-imidacloprid monoclonal antibodies as signal probes using the glutaraldehyde cross-linking method. Under the excitation of 980 nm excitation light, the fluorescence signals of the synthesized core–shell NaYF_4_:Yb@NaYF_4_:Ho and monolayer NaYF_4_:Yb,Tm upconversion nanoparticles (UCNPs) were simultaneously detected at 656 and 696 nm, respectively. Imidacloprid (IMI) and clothianidin (CLO) could compete with antigen-conjugated amino Fe_3_O_4_ magnetic nanomaterials for binding to signaling probes, thus establishing a rapid and sensitive fluorescent immunoassay for the simultaneous detection of IMI and CLO. Under optimal conditions, the LOD (*IC*_10_) and sensitivity (*IC*_50_) of IMI and CLO were (0.032, 0.028) and (4.7, 2.1) ng/mL, respectively, and the linear assay ranges were at 0.032–285.75 ng/mL and 0.028–200 ng/mL, respectively. The immunoassay did not significantly cross-react with other analogs. In fruits and vegetables such as apples, oranges, peaches, cucumbers, tomatoes, and peppers, the mean recoveries of IMI and CLO ranged from 83.33% to 115.02% with relative standard deviations (RSDs) of 1.9% to 9.2% and 1.2% to 9.0%, respectively. Furthermore, the results of the immunoassay correlate well with the high-performance liquid chromatography method used to detect the actual samples.

In the work of Chang Liu et al. (Contribution 4), a Eu^3+^-MOF-253@Au electrochemiluminescence sensor was successfully constructed for the first time by encapsulating nanogold in the metal–organic framework (MOF) backbone and pore channels, and assembling Eu^3+^ on the MOF backbone. Firstly, the introduction of nanogold overcame the weakness of MOFs, which was difficult to achieve, and enhanced its catalytic performance, followed by the modification of Eu^3+^ to confer the electrochemiluminescence performance and the function of target detection on the sensor. Moreover, carbaryl was placed in an alkaline working solution to enhance the intensity of electrochemiluminescence signals, as well as to promote the hydrolysis of carbaryl into 1-naphthol, which caused the burst of the Eu^3+^-MOF-253@Au electrochemiluminescence sensor, thereby achieving the sensitive detection of carbaryl. On this basis, the electrochemiluminescence detection conditions were optimized, the performance was analyzed, and finally, it was successfully used for the detection of carbaryl with good linearity in the range of 0.2–200 µg L^−1^ and a low LOD (0.14 µg L^−1^).

Food allergies have seriously affected some people’s quality of life, and even endangered their lives. At present, there is still no effective cure for food allergies. Avoiding the intake of allergenic food is still the most effective way to prevent allergic diseases. Therefore, it is necessary to develop rapid, accurate, sensitive, and reliable analysis methods to detect food allergens from different sources. Aptamers are oligonucleotide sequences that can bind to a variety of targets with high specificity and selectivity, and they are often combined with different transduction technologies, thereby constructing various types of aptamer sensors. In recent years, with the development of technology and the application of new materials, the sensitivity, portability, and cost of fluorescence sensing technology have been greatly improved. Therefore, aptamer-based fluorescence sensing technology has been widely developed and applied in the specific recognition of food allergens. Liping Hong et al. (Contribution 5) comprehensively reviewed the classification of major allergens and their characteristics in animal and plant foods, and summarized the preparation principles and practical applications of aptamer-based fluorescence biosensors. This article presents some strategies for the rapid and sensitive detection of allergens in food matrices.

In their study, Ziwen Zhang et al. (Contribution 6) developed a rapid fluorescent and colorimetric dual-mode detection strategy for Hg^2+^ in seafoods based on the cyclic binding of the organic fluorescent dye rhodamine 6G hydrazide (R6GH) to Hg^2+^. The luminescence properties of the fluorescent R6GH probe in different systems were investigated in detail. Based on the UV and fluorescence spectra, it was determined that the R6GH has good fluorescence intensity in acetonitrile and good selective recognition of Hg^2+^. Under optimal conditions, the R6GH fluorescent probe showed a good linear response to Hg^2+^ (R^2^ = 0.9888) in the range of 0–5 µM, with a low detection limit of 2.5 × 10^−2^ µM (S/N = 3). A paper-based sensing strategy based on fluorescence and colorimetric analysis was developed for the visualization and semiquantitative analysis of Hg^2+^ in seafoods. The LAB values of the paper-based sensor impregnated with the R6GH probe solution showed good linearity (R^2^ = 0.9875), with Hg^2+^ concentrations in the range of 0–50 µM, which means that the sensing paper could be combined with smart devices to provide reliable and efficient Hg^2+^ detection.

Additives and antibiotic abuse during food production and processing are among the key factors affecting food safety. The efficient and rapid detection of hazardous substances in food is of crucial relevance to ensure food safety. In the study by Shijie Li et al. (Contribution 7), a water-soluble quantum dot with glutathione as a ligand was synthesized as a fluorescent probe by the hydrothermal method to achieve the detection and analysis of H_2_O_2_. The detection limits were 0.61 µM in water and 68 µM in milk. Moreover, it was used as a fluorescent donor probe, and manganese dioxide nanosheets were used as a fluorescent acceptor probe in combination with an immunoassay platform to achieve the rapid detection and analysis of enrofloxacin (ENR) in a variety of foods with LODs of 0.05–0.25 ng/mL in foods. The proposed systems provide new ideas for the construction of fluorescence sensors with high sensitivity.

The study from Qi Zhang et al. (Contribution 8) proposes a facile and versatile layer-by-layer strategy without any special surface modifications for the preparation of magnetic metal-organic frameworks (MMOFs) supporting molecularly imprinted polymer nanoparticles (MMOFs@MIP), which are based on a magnetically susceptible core conjugated with an imidazole-derived self-assembled layer and a silane-based imprinted shell. Metal–organic frameworks (MOFs) with systematically tailored structures have been suggested as promising precursors to the preparation of diverse functional materials. The obtained MMOFs@MIPs, which integrated the advantages of Fe_3_O_4_, MOFs, and MIPs, were characterized and exhibited good magnetic properties, a rapid mass transfer rate, and excellent adsorption selectivity, as well as capacity for the targeted molecular—bisphenol A (BPA). Moreover, the MMOFs@MIPs were employed as adsorbents in magnetic solid-phase extraction (MSPE) to selectively bind and rapidly separate BPA from real samples, with satisfactory recovery rates ranging from 88.3% to 92.3%. More importantly, the desirable reusability of MMOFs@MIPs was also evaluated, and the recovery was maintained above 88.0% even after five re-use cycles. Furthermore, combined with high-performance liquid chromatography (HPLC) analysis, a novel MSPE-HPLC method was developed, enabling the highly selective and sensitive detection of BPA in a wide linear range of 0.5–5000 µg L^−1^, with a low LOD of 0.1 µg L^−1^. This work contributes a promising method for constructing various functional nanoparticle@MOFs@MIP hybrid materials for applications in many different fields.

Ying Guo et al. constructed a novel fluorescent molecularly imprinted nanosensor (N, S-GQDs@ZIF-8@MIP) based on the nitrogen and sulfur co-doped graphene quantum dots decorated zeolitic imidazolate framework-8 for the detection of octopamine (OA) (Contribution 9). Herein, ZIF-8 with a large surface area was introduced as a supporter of the sensing system, which effectively shortened the response time of the sensor. Meanwhile, high green luminescent N, S-GQDs and a maximum emission wavelength of 520 nm under 460 nm excitation and a 12.5% quantum yield, were modified on the surface of ZIF-8 as a signal tag that could convert the interactions between the sensor and OA into detectable fluorescent signals. Finally, N, S-GQDs@ZIF-8@MIP was acquired through the surface molecular imprinting method. Due to the synergy of N, S-GQDs, ZIF-8, and MIP, the obtained sensor not only demonstrated higher selectivity and sensitivity than N, S-GQDs@ZIF-8@NIP, but also displayed faster fluorescence responses than N, S-GQDs@MIP. Under optimal conditions, the developed sensor presented a favorable linear relationship in the range of 0.1–10 mg L^−1^, with a detection limit of 0.062 mg L^−1^. Additionally, the proposed N, S-GQDs@ZIF-8@MIP strategy was effectively applied to the detection of OA in fermented samples.

Xiaohui Wang et al. constructed a fluorescent sensor (NH_2_-UIO-66(Zr)@MIP) based on a molecularly imprinted polymer-coated amino-functionalized zirconium (IV) metal–organic framework, and initially used it for the ultrasensitive determination of oxytetracycline (Contribution 10). Developing sensitive and effective methods to monitor oxytetracycline residues in food is of great significance for maintaining public health. NH_2_-UIO-66 (Zr), with a maximum emission wavelength of 455 nm under 350 nm excitation, was prepared using a microwave-assisted heating method. The NH_2_-UIO-66(Zr)@MIP sensor with specific recognition sites for oxytetracycline was then acquired by modifying a molecularly imprinted polymer on the surface of NH_2_-UIO-66 (Zr). The introduction of NH_2_-UIO-66 (Zr) as both a signal tag and supporter could strengthen the sensitivity of the fluorescence sensor. Thanks to the combination of the unique characteristics of the molecularly imprinted polymer and NH_2_-UIO-66 (Zr), the prepared sensor not only exhibited a sensitive fluorescence response, specific identification capabilities, and a high selectivity for oxytetracycline, but also showed good fluorescence stability, satisfactory precision, and reproducibility. The fabricated sensor displayed fluorescent linear quenching in the OTC concentration range of 0.05–40 µg mL^−1^, with a detection limit of 0.012 µg mL^−1^. More importantly, the fluorescence sensor was finally applied for the detection of oxytetracycline in milk, and the results were comparable with those obtained using the HPLC approach. Hence, the NH_2_-UIO-66 (Zr)@MIP sensor possesses great application potential for the accurate evaluation of trace oxytetracycline in dairy products.

Currently, detection technologies regarding food contaminants are still facing multiple challenges, which include issues such as the effective removal of food matrices and the monitoring and control of hazardous substances generated during food processing. It is believed that the development of more efficient, accurate, and sensitive strategies for the detection and control of food contaminants will be gradually promoted with the continuous advancement of nanomaterials, food-related research, and chemometrics. The aim of this Special Issue is to publish high-quality articles on the accurate and rapid detection of various types of food contaminants in the areas of fluorescent nanomaterials, biometrics and biomimetic recognition, and fluorescent molecules based on organic dyes and quantum dots, in order to promote the further development of nanomaterial-based optical sensing and detection methods.
